# Caveolin-1 Promotes Chemoresistance of Gastric Cancer Cells to Cisplatin by Activating WNT/β-Catenin Pathway

**DOI:** 10.3389/fonc.2020.00046

**Published:** 2020-02-03

**Authors:** Xi Wang, Bin Lu, Chunyan Dai, Yufei Fu, Ke Hao, Bing Zhao, Zhe Chen, Li Fu

**Affiliations:** ^1^Key Laboratory of Digestive Pathophysiology of Zhejiang Province, The First Affiliated Hospital of Zhejiang Chinese Medicine, Zhejiang Chinese Medical University, Hangzhou, China; ^2^Department of Gastroenterology, First Affiliated Hospital of Zhejiang Chinese Medical University, Hangzhou, China; ^3^Research Center of Blood Transfusion Medicine, Ministry of Education Key Laboratory of Laboratory Medicine, Zhejiang Provincial People's Hospital, People's Hospital of Hangzhou Medical College, Hangzhou, China; ^4^Department of Anesthesiology, First Affiliated Hospital, School of Medicine, Zhejiang University, Hangzhou, China; ^5^Guangdong Provincial Key Laboratory of Regional Immunity and Diseases, Department of Pharmacology and Shenzhen University International Cancer Center, Shenzhen University School of Medicine, Shenzhen, China

**Keywords:** Caveolin-1, WNT/β-catenin pathway, chemoresistance, gastric cancer, cisplatin

## Abstract

Drug resistance is a major challenge for chemotherapy in treating human gastric cancer (GC), as the underlying molecular mechanism of chemoresistance in GC remains unknown. Caveolin-1 (Cav-1) is a scaffold protein of plasma membrane caveolae that acts as a tumor modulator by interacting with several cell signals. In this research, we showed that the survival rate of GC cells to cisplatin (CDDP) increased in the presence of Cav-1. Moreover, Cav-1 overexpression inhibited cisplatin-induced apoptosis and improved the survival rate of GC cells. Cav-1 overexpression and knock-down experiments indicated that Cav-1 expression stimulated wingless-type MMTV integration site (WNTs) pathway through the phosphorylation of LRP6 and dephosphorylation of β-catenin. Cav-1 was positively associated with the increase of WNT downstream target gene Met, which led to the activation of HER2 signaling. Moreover, our results demonstrated that the expression of Cav-1 and Met were positively associated with the resistance of GC cells to cisplatin. Collectively, Cav-1 enhances the cisplatin-resistance of GC cells by activating the WNT signaling pathway and Met-HER2 crosstalk. Understanding the role of Cav-1 in the chemoresistance of GC would help to develop novel therapies for a better treatment outcome of GC patients.

## Introduction

Gastric cancer (GC) is one of the most common types of malignancy and a leading cause of cancer death worldwide (1). Chemotherapy remains the primary method to treat patients with advanced-stage gastric cancer (2), though the treatment outcome is poor due to the resistance of GC cells to chemotherapy agents, including cisplatin (3). The underlying mechanism of cisplatin resistance in GC remains largely unknown.

Caveolae is the flask-shape vesicular invaginations of plasma membranes enriched in differentiated cells of epithelial and mesenchymal origin (4), which facilitates the localization of multiple receptors and signaling molecules (kinases, G-proteins and adhesion molecules). Caveolin-1 (Cav-1) is the essential structural protein of caveolae, and acts as a tumor suppressor by regulating the signaling transduction associated with the cell proliferation, differentiation, invasion, adhesion, apoptosis and metastasis (5). Cav-1 can regulate the members of growth-factor-activated Ras-p42/44 mitogen-activated protein kinase (MAPK) pathway, as well as proteins of the pro-survival phosphatidylinositol 3-kinase/Akt pathway (6). Cav-1 increases in multiple human drug-resistant cancer cells, such as cisplatin-resistant ovarian cancer cells (7), 5-fluorouracil-resistant colorectal cancer cells (8) and multidrug-resistant lung cancer cells (9), indicating that the resistance to the chemotherapy is tightly correlated with the upregulation of Cav-1 expression. However, the relationship between Cav-1 and chemoresistance in gastric cancer remains unknown.

The wingless-type MMTV integration site (WNT) family constitutes a series of cysteine-rich secreted proteins implicated in mediating a wide variety of physiological and pathological processes. In the canonical WNT pathway, WNTs ligand binds to its receptor complex which consists of a seven-pass transmembrane receptor, frizzled (FZD), a single-pass transmembrane receptor, and low-density lipoprotein receptor-related protein (LRP5/6), to stimulate the signaling pathway. Phosphorylation of LRP5/6, with the assistance of Disheveled (Dvl) can facilitate the interaction of the destruction complex (formed by APC, Axin, CK1a, and GSK-3β) with the cytoplasmic tail of LRP, which prevents the phosphorylation and ubiquitin-mediated degradation of β-catenin. On this respect, the free β-catenin accumulates in the cytoplasm and is translocated into the nucleus where it activates the canonical WNT-triggered gene transcription along with the transcription complex TCF/LEF (10). Accumulating evidences showed that WNT pathway had diverse functions in regulating cell proliferation, migration, inflammation, fibrosis and carcinogenesis (11, 12). Recent studies demonstrated that WNT6 as a novel target gene of Cav-1 enhanced the resistance of human GC cells to anthracycline drugs (13). However, the mechanism of Cav-1 in the regulation of WNT pathway and the related chemoresistance in gastric cancer cells has not been fully understood.

Here, we investigated the function of Cav-1 in the activation of the canonical WNT pathway, and its relationship to cisplatin-resistance of GC cells. We observed that Cav-1 enhanced the resistance of human GC cells to apoptosis induced by cisplatin via augmenting WNT signaling and Met-HER2 pathway. Our study revealed a novel role of Cav-1 as a critical factor in promoting WNT-dependent anti-apoptosis signaling, which facilitated GC cell chemoresistance. These results support the notion that targeting Cav-1 may be a valuable alternative to develop new therapies for GC patients.

## Materials and Methods

### Cell Culture

Human gastric cancer cell lines AGS and MGC803 were obtained from the Typical Cell Culture Collection Committee of Chinese Academy of Sciences (Shanghai, China). AGS cells were grown in Ham's F-12 Nutrient Mixture medium (Gibco, Thermo Fisher Scientific, Inc., Waltham, MA, USA), and MGC803 cells were cultured in RPMI-1640 medium (Gibco, Thermo Fisher Scientific, Inc.) supplemented with 10% fetal bovine serum (Gibco, Thermo Fisher Scientific, Inc.) in a 5% incubator at 37°C. AGS cells were naturally devoid of Cav-1, while MGC803 cells expressed endogenous Cav-1. Human cisplatin-resistance gastric cancer cell lines AGS/CDDP were obtained from the parental AGS cells which were exposed to cisplatin with gradient concentration ranging from 1 to 9 μg/ml.

### Cell Transfection

Cav-1-related plasmid vectors were all constructed by Guangzhou FulenGen Co., Ltd. (Guangzhou, Guangdong, China). The Met shRNA plasmid vector (shMet) was purchased from GeneChem Co., Ltd. (Shanghai, China). All plasmid vectors were transfected into GC cells using HieffTransTM Liposomal Transfection Reagent (Yeasen Biotechnology Co., Ltd., Shanghai, China) according to the manufacture's instruction. For the overexpression of Cav-1, AGS cells were transfected with 2 μg Cav-1-overexpression plasmid (Cav-1+) or its empty vector (EV) in a 6-well plate for 24, 48, and 72 h, respectively. To ablate endogenous Cav-1 expression, MGC803 cells were cultured in a 6-well plate at 3 × 10^5^ cells/plate. After 18 h, cells were transiently transfected with 2 μg Cav-1 shRNA (shCav-1) or a negative control shRNA (NC) for 24, 48, and 72 h, respectively. To explore the interaction between Cav-1 and Met, AGS cells were simultaneously transfected with Cav-1-overexpression and shMET plasmid vectors for 72 h. Then, the cells were washed with PBS and harvested for real-time PCR and/or western blotting analysis. The inserted human Cav-1 (Gene ID:857) shRNA sequence was 5′-gcaatgtccgcatcaacttgc-3′, while the target sequence of shMet (Gene ID: 4233) was 5′-ctggtggcactttacttactt-3′.

### Cell Survival Assay

Cells were plated and grown in 96-well for 18 h. Then, AGS cells were transfected with Cav-1-overexpression plasmid or its empty vector for 24 h, while MGC803 cells were transfected with shCav-1 plasmid or its negative control shRNA for 48 h followed by treatment with cisplatin for another 24 h. AGS cells were transfected with shMet plasmid for 24 h and then treated with cisplatin for another 72 h. The number of viable cells was determined by a microplate spectrophotometer using appropriate CCK-8 at 450 nm using.

#### Real-Time PCR

Total RNAs were extracted from cell lines using General RNA Extraction Kit (Guangzhou Dongsheng Biotech Co., Ltd., Guangzhou, Guangdong, China) according to the manufacturer's instruction. Total RNA was reverse-transcribed to cDNA usingPrimeScript^TM^ RT Master Mix (Takara Biomedical Technology Co., Ltd., Beijing, China). Changes in human Cav-1 (NM_001753.5) and Met (NM_001324401.2) were performed using SYBR®*Premix EX Taq*^TM^ (Takara Biomedical Technology Co., Ltd., Beijing, China) on Applied Biosystems 7900 Real-Time PCR System (ABI, Foster City, CA, USA). Human β-actin (NM_001101.3) was amplified for each sample as an endogenous control, and the cycle number at threshold (CT value) of each target was normalized by subtracting the CT value of β-actin. Primer sequences are listed as following: Cav-1 sense, 5′ CAGAACCAGAAGGGACACAC 3′ and antisense, 5′ AAAGAGGGCAGACAGCAAGC 3′; Met sense, 5′ TTCCTGGGCACCGAAAGATAAA 3′, and antisense, 5′ GCACCAAGGAAAATGTGATGCT 3′, β-actin sense, 5′ GAAGGATTCCTATGTGGGCG 3′, and antisense, 5′ TCATTGTAGAAGGTGTGGTG 3′.

### Annexin V-PE/7-AAD Double Staining Assay

For flow cytometry analysis of annexin V externalization, cells were seeded on a 6-well plate (2 × 10^5^ cells per well) to achieve 70% confluence overnight before assay. AGS cells were transfected with Cav-1 overexpression vector or empty vector for 24 h, followed by incubation with 20 μg/ml cisplatin for 12 and 24 h, respectively. MGC803 cells were transfected with shCav-1 vector or negative control vector for 48 h, followed by incubation with 8 μg/ml cisplatin for 12 and 24 h, respectively. Annexin V levels were determined using an Annexin V-PE/7-AAD staining kit (BD Pharmingen, San Diego, CA, USA) according to the manufacturer's protocol. All cells were analyzed by flow cytometry using a BD FACSCanto II flow cytometry (BD Pharmingen) to detect the fluorescence signal.

### Western Blot

All cell proteins were loaded onto 10% SDS-PAGE gels and transferred to PVDF members. Blots were blocked for 1 h in TBST containing 5% nonfat dry milk. Then, membranes were incubated with primary antibodies at 4°C overnight. The following primary antibodies were used: monoclonal rabbit anti-Caveolin-1 (1:10,000), monoclonal rabbit anti-β-catenin Ser33/37 (1:500) (Abcam, Cambridge, UK) and the other antibodies (1:1,000) (Cell Signaling Technology, Beverly, MA, USA).

### Immunofluorescence Analysis

AGS cells were grown to ~60% on 14–mm sterile glass coverslips in 24-well plates for 16 h, and then were transfected with Cav-1-overexpression plasmid or its empty vector for 24 h. After the medium was removed, the cells were washed with 1 × PBS and fixed with 4% formaldehyde for 15 min at room temperature. After incubated with ice-cold 100% methanol for 10 min at −20°C, the cells were blocked with 5% BSA for 1 h and went through immunostaining. The cells were treated with primary antibodies overnight at 4°C, followed by incubation with secondary antibody for 1 h at room temperature. Subsequently, the coverslips were stained and mounted with DAPI Fluoromount-G® (SouthernBiotech, Bimingham, AL, USA) for 10 min. The following primary antibodies were used: rabbit anti-Caveolin-1 (1:400), mouse anti-β-catenin (1:200) (Cell Signaling Technology, Beverly, MA, USA).

### TOP/FOP Flash Luciferase Assay

TOP/FOP flash luciferase assay was used to detect the transcriptional activity of the TCF-dependent WNT/β-catenin signaling. The reporter plasmid of β-catenin (TOP Flash) and its mutant control (FOP Flash) were from Dr. Fu (Shenzhen University, China). AGS cells were grown in a 24-well plate (5 × 10^4^ cells per well) overnight and transiently transfected with Cav-1 overexpression vector or empty vector for 24 h. Subsequently, the cells were co-transfected with TOP or FOP flash luciferase plasmids and pRL-TK vector (also from Dr Fu) for 24 h. The activity of TOP/FOP flash reporter was examined by Promega Dual-Luciferase Reporter Assay System, and the β-catenin-driven transcription was presented as the TOP/FOP ratio which was normalized to the relative ratio of Renilla Luciferase activity, respectively.

### Statistical Analysis

The difference between treatments was analyzed with two-tailed paired *t*-test using SPSS version 16.0 (SPSS Inc., Chicago, IL, USA). All values are given as mean± the standard error of the mean (SEM) from at least three independent cell experiments. A value of ^*^p < 0.05 was considered statistically significant.

## Results

### Cav-1 Promotes Resistance of Human GC Cells to Cisplatin

**Figure 1:**
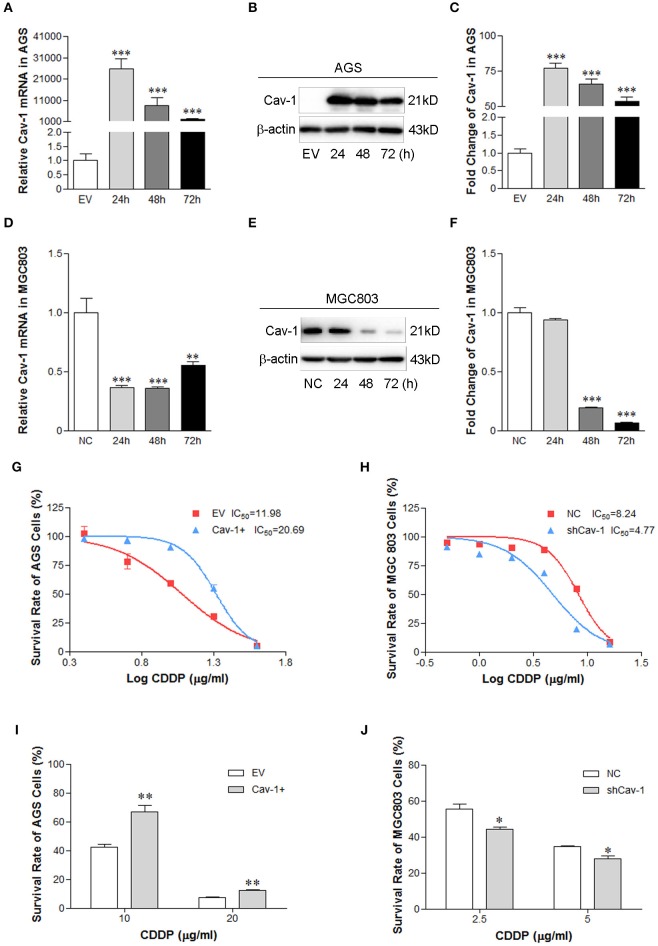
Cav-1 induces the survival of GC cell lines in the presence of cisplatin chemotherapy. **(A)** The mRNA expression level of Cav-1 was significant up-regulated in Cav-1-transfected AGS cells compared with control cells. **(B)** The protein level was in consistent with the mRNA expression of Cav-1. **(C)** The relative protein level of Cav-1 in AGS cells was analyzed. **(D)** The endogenous expression of Cav-1 mRNA in MGC 803 cells was mostly inhibited after the cells were transfected with shCav-1 vector for 24 h. **(E)** The protein level of Cav-1 was decreased after the cells were transfected with shCav-1 for 48 h. **(F)** The relative protein level of Cav-1 in MGC803 cells was analyzed. **(G–J)** Cav-1-overexpression or –repression GC cells were treated with increasing concentrations of cisplatin for 24 h. Cell viability was assessed by CCK-8 assay. AGS/Cav-1+ and MGC803/NC cells were more resistant than AGS/EV **(G)** and MGC803/shCav-1 cells **(H)**. The survival of AGS/Cav-1+ cells was increased in the presence of cisplatin at the concentration of 10 and 20 μg/ml **(I)**. The survival of MGC803/shCav-1 cells was decreased in the presence of cisplatin at the concentration of 2.5 and 5 μg/ml **(J)**. The fold change of protein was standardized according to the protein levels in the EV or NC group. Optical density (OD) values are calculated as % survival ± SEM (*n* = 3) of controls. Data are shown as mean ± SEM (*n* = 3). *P < 0.05, **P < 0.01, ***P < 0.001 compared with the empty vector or negative control. CDDP, cisplatin.

Growing evidences have shown that upregulation of Cav-1 was found in multiple drug-resistance cancer cells (7–9). To explore the function of Cav-1 in mediating drug resistance of GC cells, AGS cells were transiently transfected with Cav-1 (Cav-1+) or empty vector (EV) for 24, 48, and 72 h, respectively. We found that AGS cells transfected with Cav-1 for 24 h had higher levels of Cav-1 mRNA (Figure 1A) and protein than those transfected with EV for 24 h (Figures 1B,C). The endogenous Cav-1 was knocked down by transient transfection of shCav-1 (shCav-1) or negative control shRNA (NC) in MGC803 cells for 24, 48, and 72 h, respectively. Results from real-time PCR showed the mRNA expression of Cav-1 was significantly reduced in MGC803 cells after the cells were transfected with shCav-1 for 24 and 48 h (Figure 1D), while the protein level of Cav-1 decreased at hours 48 and 72 after transfection (Figures 1E,F). AGS/Cav-1+ cells and MGC803/shCav-1 cells were then exposed to cisplatin for 24 h. Concentration response curves in CCK-8 assays showed that AGS/Cav-1+ cells were more resistant than EV clones and the IC50 increased significantly from 11.98 to 20.69 (Figure 1G), while MGC803/shCav-1 cells were more sensitive to cisplatin than control cells, and the IC50 dropped from 8.24 to 4.77 (Figure 1H). Based on the IC50 value of each GC cell line, AGS cells were treated with 10 and 20 μg/ml cisplatin respectively for 24 h, while MGC803 cells were exposed to 2.5 and 5 μg/ml cisplatin, respectively, for 24 h. We found that AGS/Cav-1+ cells showed a significant decrease in cisplatin-induced cell death in compared with the control cells (Figure 1I). However, transient transfection of shCav-1 into MGC803 cells decreased cell survival rate in the present of cisplatin as compared with control cells (Figure 1J).

### Cav-1 Inhibits the Cisplatin-Induced Apoptosis in GC Cells

**Figure 2:**
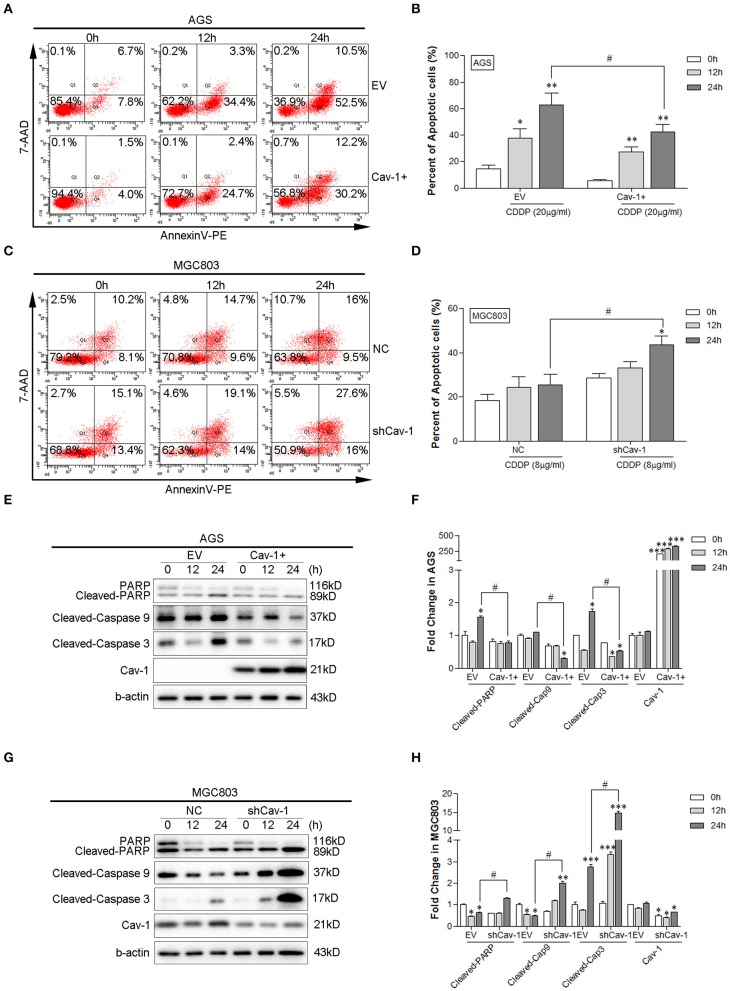
Cav-1 attenuates apoptotic response induced by cisplatin in GC cells. **(A)** Overexpression of Cav-1 inhibited the apoptosis of AGS cells. **(B)** Statistical analysis of the apoptotic rate of AGS cell population after CDDP treatment. **(C)** Knock-down of Cav-1 enhanced the apoptosis of MGC803 cells. **(D)** Statistical analysis of the apoptotic rate of MGC803 cell population after CDDP treatment. **(E)** The expression of cleaved caspase-3, cleaved caspase-9, and cleaved PARP were notably down-regulated in AGS/Cav-1+ cells compared with AGS/EV cells. **(F)** The relative levels of cleaved caspase-3, cleaved caspase-9, cleaved PARP and Cav-1 in AGS cells were analyzed. **(G)** Knock-down of Cav-1 substantially increased the levels of cleaved caspase-3, cleaved caspase-9, and cleaved PARP in MGC803/shCav-1 cells compared with MGC803/NC cells. **(H)** The relative levels of cleaved caspase-3, cleaved caspase-9, cleaved PARP, and Cav-1 in MGC803 cells were analyzed. Data are shown as mean ± SEM (*n* = 3). *P < 0.05, **P < 0.01, ***P <0.001 compared with AGS/EV cells or MGC803/NC cells. ^#^*P* <0.05 compared with CDDP for 24 h after transfected with the empty or negative control vector. CDDP, cisplatin.

Inhibition of cell apoptosis is a common mechanism that induces the drug-resistance of cancer cells. Thus, we determined whether Cav-1 could regulate the cisplatin-induced apoptosis in GC cells. Using Annexin V-PE/7-AAD flow cytometry assay, we found the level of apoptosis meaningfully declined with in the presence of Cav-1 after exposed to 20 μg/ml cisplatin for 24 h (Figures 2A,B). Whereas, in the absence of Cav-1, the gastric cell apoptosis remarkably increased with 8 μg/ml cisplatin for 24 h (Figures 2C,D). The expression of apoptosis-related proteins in both AGS and MGC803 cells were further evaluated. Cisplatin enhanced apoptotic response in the cleavage of caspase-3, caspase-9, and PARP in both AGS and MGC803 cells. Cav-1 overexpression inhibited this positive enhancement by cisplatin on cleavedcaspase-3, cleaved caspase-9 and cleaved PARP (Figures 2E,F), while the lack of Cav-1 remarkably improved the level of cleaved caspase-3, cleaved caspase-9, and cleaved PARP (Figures 2G,H). These results support the notion that Cav-1 could act as a critical factor to attenuate apoptosis and enhance the resistance of chemotherapeutic agent cisplatin in human GC cells.

### Cav-1 Promotes the Activation of WNT/β-Catenin Signaling

**Figure 3:**
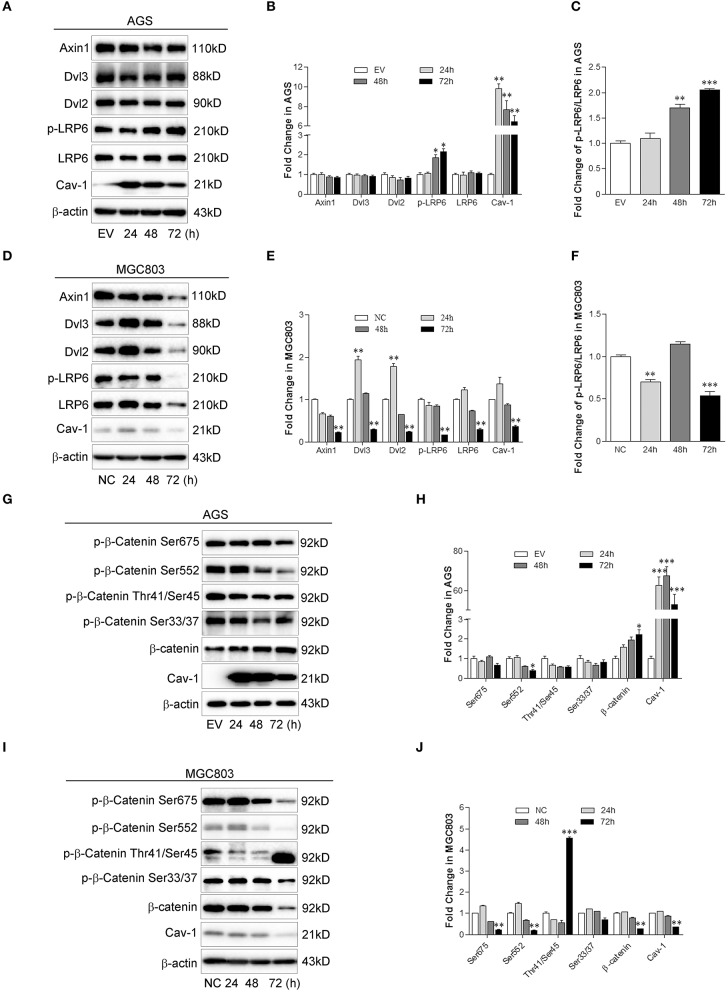
Cav-1 regulates the activation of key molecules involved in canonical WNT signaling pathway. **(A)** Overexpression of Cav-1 increased the phosphorylation of LRP6 but not the expression of Axin1, Dvl2, Dvl3, and total LRP6 in AGS/Cav-1+ cells compared with AGS/EV cells. **(B)** The relative levels of Axin1, Dvl2, Dvl3, p-LRP6, total LRP6, and Cav-1 in AGS cells were analyzed. **(C)** The fold change of p-LRP6 relative to total LPR6 in AGS cells was analyzed. **(D)** Knock-down of Cav-1 obviously inhibited the phosphorylation of LRP6, as well as expression of Axin1, Dvl2, Dvl3, and total LRP6 in MGC803/shCav-1 cells compared with MGC803/NC cells. **(E)** The relative levels of Axin1, Dvl2, Dvl3, p-LRP6, total LRP6, and Cav-1 in MGC803 cells were analyzed. **(F)** The fold change of p-LRP6 relative to total LPR6 in MGC803 cells was analyzed. **(G)** Following the activation in the upstream molecules of β-catenin, the phosphorylation of β-catenin at Ser552 site was notably reduced, while total β-catenin was increased in AGS/Cav-1+ cells compared with AGS/EV cells. **(H)** The relative levels of p-β-catenin at Ser33/37, Tyr41/Ser45, Ser552, and Ser675 sites, total β-catenin and Cav-1 in AGS cells were analyzed. **(I)** Knock-down of Cav-1 obviously increased phosphorylation of β-catenin at Thr41/Ser45 sites but decreased total β-catenin in MGC803 cells, although there was a little effect on the dephosphorylation of β-catenin at Ser33/37, Ser552, and Ser675. **(J)** The relative levels of p-β-catenin at Ser33/37, Tyr41/Ser45, Ser552, and Ser675 sites, total β-catenin and Cav-1 in MGC803 cells were analyzed. Data are shown as mean ± SEM (*n* = 3). *P < 0.05, **P < 0.01, ***P <0.001 compared with the EV or NC group.

Recent evidence showed that Cav-1-induced WNT6 activation enhances the resistance of GC cells to anthracycline drugs (13). Canonical WNT signaling may promote the survival and proliferation of cancer cells and facilitate cellular chemoresistance (12). To further explore the relationship between Cav-1 and canonical WNT signaling, we analyzed the protein level of several key molecules involved in canonical WNT signaling. We found that the phosphorylation of LRP6 (p-LRP6) (Figures 3A,B) and the ratio of p-LRP6 and total LRP6 (Figure 3C) were both increased in the present of Cav-1. However, there was no difference in total LRP6, as well as Axin1, Dvl2, or Dvl3, a component of the destruction complex (Figures 3A,B). In the absence of Cav-1, the protein expression of p-LRP6, LRP6, Axin1, Dvl2, and Dvl3 were meaningfully inhibited (Figures 3D,E), and the expression of p-LRP6 was also decreased compared with that of total LRP6 (Figure 3F).

In canonical WNT signaling, phosphorylated β-catenin is recognized by a dedicated E3 ubiquitin ligase complex and ubiquitinated by proteasome. Dephosphorylation of β-catenin triggers the cytoplasmic accumulation of β-catenin and promotes the downstream signaling induced by WNTs (10). As shown in AGS/Cav-1+ cells, the level of phosphorylated β-catenin (p-β-catenin) at Ser552 was notably decreased, whereas the total β-catenin levels were increased due to Cav-1 overexpression. In addition, Cav-1 showed no impact on the level of p-β-catenin at Ser33/37, Thr41/Ser45, and Ser675 (Figures 3G,H). Although the reduction of Cav-1 expression increased the dephosphorylation of β-catenin at Ser33/37, Ser552, and Ser675, Cav-1 knock-down improved the level of p-β-catenin at Thr41 and Ser45, and attenuated the accumulation of β-catenin (Figures 3I,J). These data indicate that Cav-1 may be a positive regulator of β-catenin activation related to the resistance of cisplatin.

### Cav-1 Modulates the Expression of Target Genes via Canonical WNT Pathway

**Figure 4:**
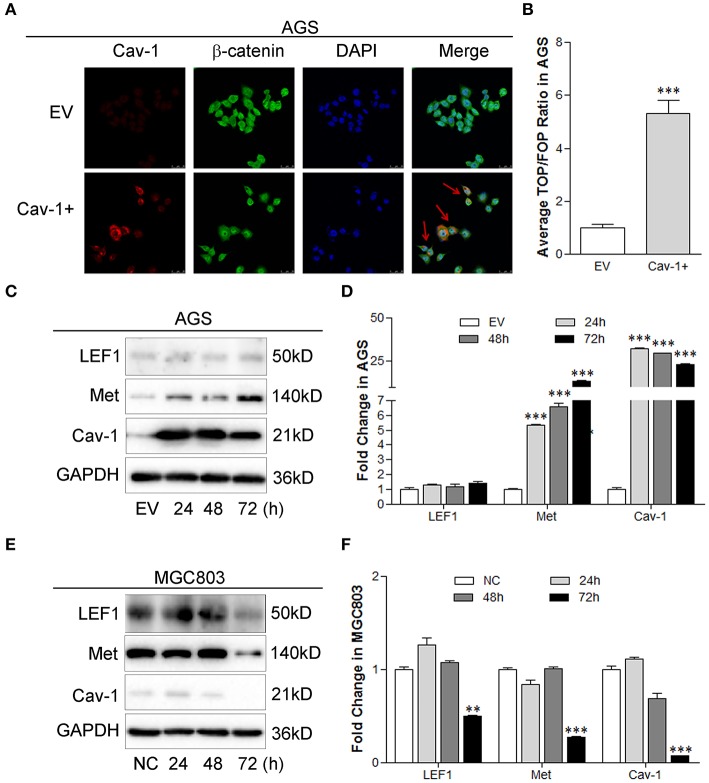
Cav-1 induces the expression of target genes by activating the β-catenin pathway. **(A)** The immunofluorescence images showed that more β-catenin was located in the nucleus after transfected with Cav-1 for 72 h (as shown in red arrow). **(B)** TOP/FOP flash luciferase analysis showed that the level of β-catenin-LEF/TCF transcription was remarkably elevated by the overexpression of Cav-1. **(C)** The expression of Met was significantly increased in AGS cells at 24 h post Cav-1 transfection, while Cav-1 didn't affect the expression of LEF1. **(D)** The relative levels of LEF1, Met, and Cav-1 in AGS cells were analyzed. **(E)** The protein levels of LEF1 and Met were both reduced in Cav-1-repressed MGC803 cells at 72 h post Cav-1 transfection. **(F)** The relative levels of LEF1, Met, and Cav-1 in MGC803 cells were analyzed. Data are shown as mean ± SEM (*n* = 3). **P < 0.01, ***P <0.001 compared with the EV or NC group.

The β-catenin-mediated target gene transcription has been identified as one of the important function related to the activation of canonical WNT/β-catenin pathway. To identify whether Cav-1 could modulate the target gene transcription induced by β-catenin, we examined the nuclear localization of β-catenin, the transcriptional activity of the TCF-dependent β-catenin, and the expression of known canonical targets including LEF1 and Met. Our results showed that overexpression of Cav-1 facilitated the migration of β-catenin to the nucleus (Figure 4A) and enhanced the transcriptional activity of β-catenin-LEF/TCF (Figure 4B). We also found that Cav-1 overexpression elevated the expression of Met, but had no impact on LEF1 expression in AGS cells (Figures 4C,D). In MGC803 cells, repression of Cav-1 notably inhibited the protein level of LEF1 and Met (Figures 4E,F). These data suggest that the canonical WNT/β-catenin pathway may be involved in the Cav-1-mediated chemoresistance of GC cells by targeting the Met.

### Cav-1 Induces the HER2 Expression by Activating Met in GC Cells

**Figure 5:**
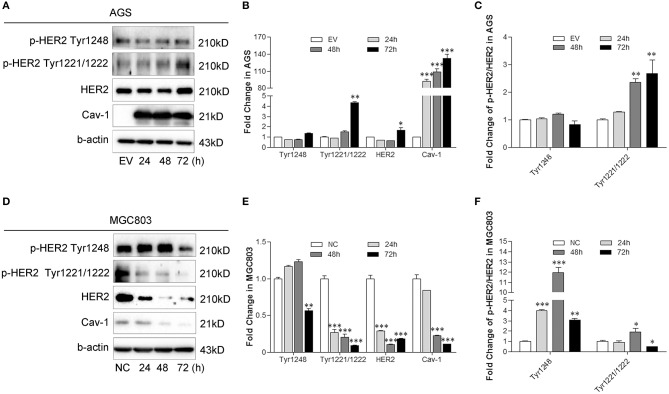
Cav-1 induces the activation of HER2 in GC cells. **(A)** Cav-1 stimulated the phosphorylation of HER2 at Tyr1221/1222 sites, but had no effect on the Tyr1248 phosphorylation in AGS/Cav-1+ cells compared with AGS/EV cells. **(B)** The relative levels of p-HER2 at Tyr1221/1222 and Tyr1248 sites, total HER2 and Cav-1 in AGS cells were analyed. **(C)** The fold change of p-HER2 relative to total HER2 in AGS cells was analyzed. **(D)** Both total and phosphorylated HER2 expression levels were attenuated in MGC803/shCav-1 cells compared with MGC803/NC cells. **(E)** The relative levels of p-HER2 at Tyr1221/1222 and Tyr1248 sites, total HER2 and Cav-1 in MGC803 cells were analyzed. **(F)** The fold change of p-HER2 relative to total HER2 in MGC803 cells was analyed. Data are shown as mean ± SEM (*n* = 3). *P < 0.05, **P < 0.01, ***P <0.001 compared with the EV or NC group.

Human epidermal growth factor receptor-2 (HER2) is abnormally expressed in gastric cancer and is closely associated with cancer chemoresistance. Inhibition of HER2-expression significantly increases the sensitivity of cells to chemotherapeutic drugs (14). A positive crosstalk exists between Met and HER2, and both of them increase the resistance of GC cells to cisplatin (15). To elucidate the link between Cav-1 and Met-HER2 signaling, we measured the activation of HER2 with or without Cav-1 expression. Total HER2 expression and the phosphorylation of HER2 increased at Tyr1221 and Tyr1222 with Cav-1 overexpression, but there was no difference in the phosphorylation of HER2 at Tyr1248 (Figures 5A,B). Cav-1-transfected cells showed a higher level of p-HER2 at Tyr1221/1222 site, but not at Tyr1248 site compared with control cells (Figure 5C). With the knockdown of Cav-1, there was a significant decrease in both the expression and the phosphorylation of HER2 (Figures 5D,E). Meanwhile, Cav-1 silencing led to a lower phosphorylation level of HER2 at Tyr1221/1222 site, but a higher phosphorylation level at Tyr1248 compared with control cells (Figure 5F).

**Figure 6:**
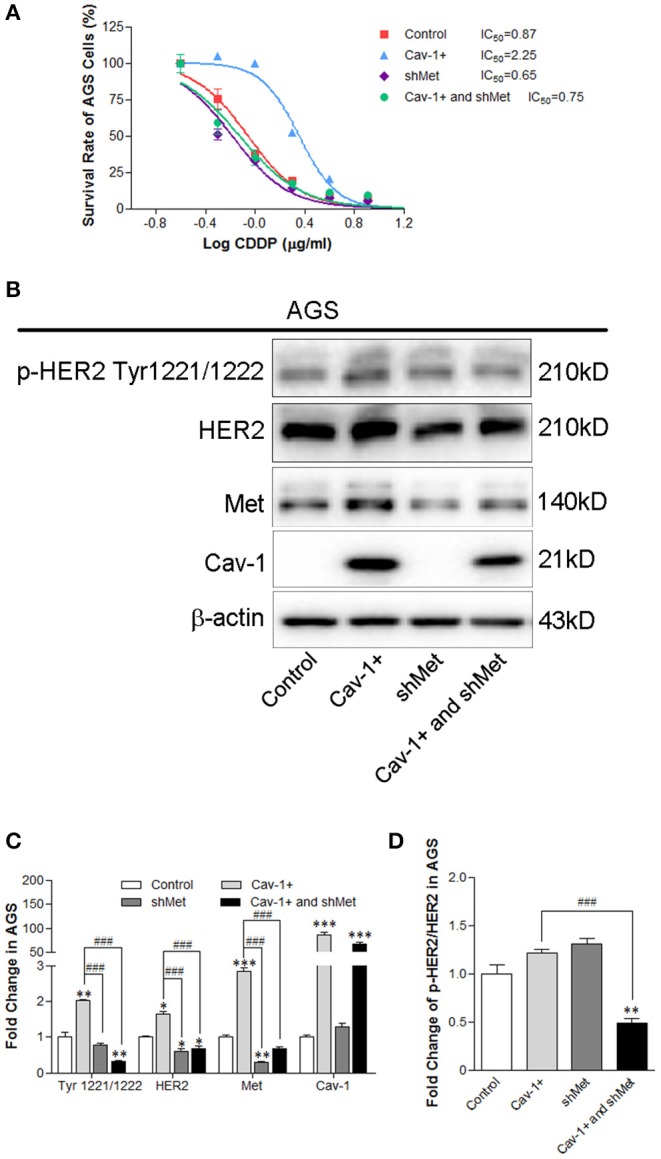
Knock-down of Met prevents the Cav-1-induced cisplatin resistance of AGS cells by targeting the Met and HER2 pathway. **(A)** The viability of AGS/Cav-1+ cells was elevated compared with AGS cells, while AGS/shMet cells were more sensitive to cisplatin than AGS/Cav-1+ cells. **(B)** Cav-1 stimulated the activation of Met, p-HER2 at Tyr1221/1222 site and total HER2 in AGS cells. Met silencing inhibited the increase of p-HER2 at Tyr1221/1222 site and total HER2 induced by Cav-1. **(C)** The relative levels of p-HER2 at Tyr1221/1222, total HER2 and Cav-1 in AGS cells were analyzed. **(D)** The fold change of p-HER2 relative to total HER2 in AGS cells was analyzed. *P < 0.05, **P < 0.01, ***P < 0.001 compared with control group. ^*###*^*P* <0.001 compared with Cav-1 overexpression group. CDDP, cisplatin.

To further investigate the interaction between Met and HER2 induced by Cav-1, we silenced the expression of Met combined with the Cav-1 overexpression and measured the effect on the cell viability, and the expression and phosphorylation level of HER2. The results showed that the inhibition of Met reversed the Cav-1-induced cisplatin-resistance of GC cells (Figure 6A) and reduced the expression of total and phosphorylation of HER2 at Try1221/1222 site in the presence of Cav-1 (Figures 6B–D). These results reveal that Cav-1 could regulate the positive interaction between Met and HER2 by activating the phosphorylation of HER2.

### Cisplatin-Resistant Gastric Cells Show Increased Expression of Cav-1 and Met

**Figure 7:**
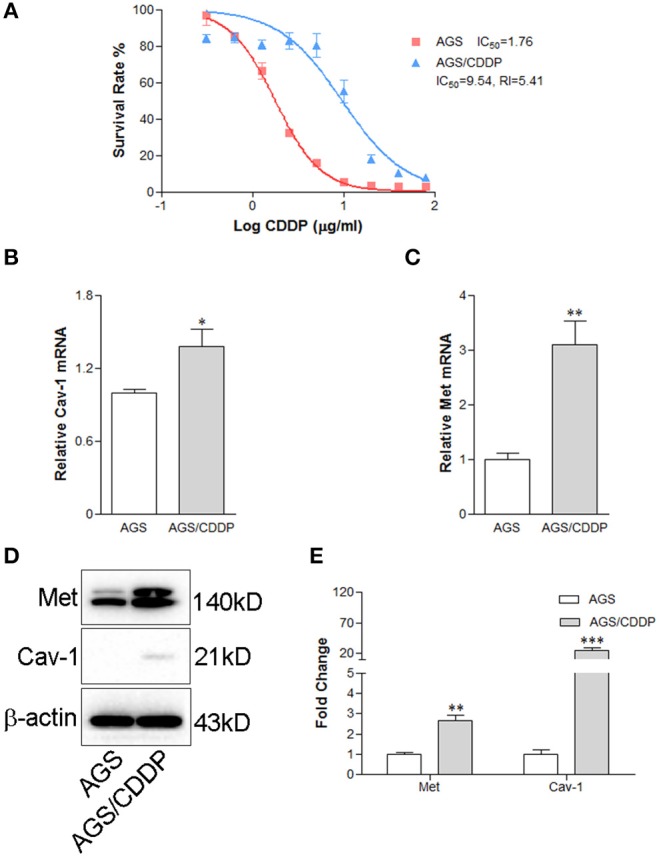
Cav-1-dependent increase of Met leads to the cisplatin-resistance of gastric cancer cells. **(A)** The cell survival rate of AGS/CDDP cells was elevated compared with AGS cells. AGS/CDDP cells showed stronger anti-cisplatin capability than their parental cells. **(B)** AGS/CDDP cells showed a higher level of Cav-1 mRNA compared with control cells. **(C)** Met mRNA expression was markedly increased in AGS/CDDP cells. **(D)** The protein levels of Cav-1 and Met were in consistent with the mRNA expression in AGS cells. **(E)** The relative levels of Met and Cav-1 in AGS and AGS/CDDP cells were analyzed. Data are shown as mean ± SEM (*n* = 3). *P < 0.05, **P < 0.01, ***P <0.001 compared with AGS cells. CDDP, cisplatin.

To explore whether the interaction between Cav-1 and Met involved in determining the drug response of GC cells, we evaluated the expression of Cav-1 and Met in cisplatin-resistant GC cells. First of all, we measured the cell survival rates of AGS cells and cisplatin-resistant gastric cells, AGS/CDDP. As shown in Figure 7A, the IC_50_ values of AGS/CDDP cells and their parental cells were 1.76 and 9.54 μg/ml, respectively, and the Resistance Index (RI) was 5.41. Meanwhile, AGS/CDDP cells were more resistant than their parental cells. Consistently, both the mRNA (Figures 7B,C) and protein expression (Figures 7D,E) of Cav-1 and Met were significantly increased in AGS/CDDP cells. The above data demonstrate that Cav-1-induced activation of Met could be closely related with the cisplatin resistance of GC cells.

## Discussion

Caveolae provide the physical interaction and compartmentalization of several signaling molecules. Cav-1 is the major protein component of caveolae and plays an important roles in tumorigenesis and metastasis (16). Previous studies have indicated that Cav-1 functions as a tumor suppressor and pro-apoptotic protein in the early transformation and development of several human cancer types including lung, colon, ovarian, breast cancer and osteosarcomas (6, 17–19). However, in recent years, some studies have reported that Cav-1 could exert an anti-apoptotic potential, enhances the tumor cell migration and invasion, and improves the chemoresistance (13, 20–23). After analyzing the gastric adenocarcinoma expression data downloaded from TCGA database via Genomic Data Commons Data Portal, we found that high expression of Cav-1 was associated with lower survival of GC patients (24). In this study, we aimed to determine whether Cav-1 contributed to the chemoresistance of GC cells to cisplatin and to further investigate the mechanism underlying its response to cisplatin treatment in GC cells. We found that overexpression of Cav-1 could significantly improve the tumor cell survivals after cisplatin treatment, while GC cells were more sensitive to cisplatin in the absence of Cav-1. These results illuminate that Cav-1 may be involved in promoting the resistance of GC cells to chemotherapeutic drugs, such as cisplatin.

Most of anticancer therapies, including drugs and chemotherapy, target cancer cells by promoting apoptosis. If cancer cells are chemoresistant, the apoptotic cells induced by anticancer therapy are limited. Therefore, apoptosis resistance is one of the important pathways in eliminating the chemoresistance of cancer (25). Our results showed that high level of Cav-1 obviously reduced the apoptosis ratio and decreased the expression of cleaved caspase-3, cleaved caspase-9 and cleaved PARP induced by cisplatin. However, knock-down of Cav-1 apparently enhanced the apoptosis ratio and increased the cleavage level of caspase-3, caspase-9, and PARP after cisplatin treatment. These data confirm that the induction of cisplatin-resistance by Cav-1 could suppress GC cell apoptosis, leading to higher tumor cell survival and further malignant progression.

It is well-documented that WNT/β-catenin pathway involves in a wide variety of cellular processes, such as cell growth, cell cycle, differentiation, tumorigenesis and chemoresistance (10, 13, 26, 27). Recent evidence has showed that WNT/β-catenin pathway serves as a good biomolecular target for GC treatment (28). β-catenin protein normally forms a stable complex with the APC, Axin, CK1a, and GSK-3β. Once binding to WNT ligand, Axin and APC facilitate β-catenin phosphorylation in CK1 and GSK3b by tagging those proteins for degradation by the proteasome (29). Phosphorylated LRP5/6 triggers an intracellular signaling cascade, leading to the translocation of active β-catenin to the nucleus (10). Our results showed that Cav-1 could lead an increased phosphorylation of LRP6 and dephosphorylation of β-catenin at Ser552 site, which resulted in an upregulation of active β-catenin expression. Knock-down of Cav-1 led to the dephosphorylation of LRP6 and an obvious increase of phosphorylation at Thr41/Ser45 given rise to proteasomal degradation of β-catenin. Although the change of Cav-1 stimulated the dephosphorylation of β-catenin at Ser33/37, Ser552, and Ser675 sites, they failed to impact β-catenin expression. These results suggest that Cav-1-mediated alteration of LRP6 contributes to WNT signaling by modulating phosphorylation status of β-catenin and its ubiquitin-mediated degradation, since Thr41/Ser45 are phosphorylated and Ser552 is dephosphorylated.

Non-phosphorylated β-catenin can be translocated to nucleus, where it interacts with the TCF/LEF1 transcription complex to promote transcription of WNT downstream target genes, including Met (26). MET is a proto-oncogene receptor tyrosine kinase (Met), which is also known as hepatocyte growth factor receptor. It is vital for the proliferation, survival, and invasion of cancer cells. It is noteworthy that Met is aberrantly over-expressed in human cancer cells and its overexpression is associated with an aggressive phenotype (30–32). Activated Met pathway also plays a key role in intrinsic and/or acquired drug resistance of gastrointestinal cancers (33). An elevated Met expression, as a biomarker, has been associated with a poor prognosis in patients with gastrointestinal cancer (34). Until now, there has been no report on the correlation between Cav-1 and Met in the clinical research. Here we showed that the expression of Met was related to the change of Cav-1 in GC cells. Our results proved this positive role of Cav-1 in the activation of WNT pathway by promoting Met expression, and this effect was related to the dephosphorylation of β-catenin at Ser552 site. Meanwhile, we also showed that the inhibition of Met sensitized Cav-1-expressing AGS cells to cisplatin. These results confirmed that the resistance of GC cells to cisplatin is tightly associated with the overexpression of Met induced by Cav-1. Our study provides the first evidence that Cav-1 acts as a modulator in activating the WNT/β-catenin pathway in GC cells, which would facilitate the development of new treatments for cisplatin resistance of GC patients by targeting the Met expression.

HER2 plays an important role in promoting cell proliferation and suppressing apoptosis, and its overexpression has been reported in a variety of tumors, including gastric cancer, breast cancer, ovarian cancer and non-small cell lung cancer (35). A previous study demonstrated that patients with intestinal-type and well-differentiated gastric cancer have a higher rate of HER2 positivity than patients with diffuse-type and poorly-differentiated cancer (36). Importantly, the HER2-positive status was associated with a poor prognosis and other relevant clinicopathological characteristics (37). A recent study provides the evidence that the inhibition of HER2 can significantly reverse the drug-resistance of gastric cancer cells (14). Furthermore, a positive relationship between the expression levels of Met and HER2 has been observed. When the Met expression is downregulated, the HER2 expression is decreased accordingly. Conversely, alterations in the expression of HER2 significantly reduce the Met expression (15). It shows that the chemoresistance of gastric cancer is due to the enhancement of this positive crosstalk between Met and HER2 in the survival signaling. In this study, we found that Cav-1 was positively correlated with the change of the expression of Met and phosphorylation of HER2 at Tyr1221 and 1222 sites in GC cells. Our results also showed that the increase of Met was occurred even when the Cav-1 was over-expressed for 24 h, while the phosphorylation of HER2 was observed after Cav-1 was up-regulated for 72 h. These results indicate that the activation of Met is earlier than the phosphorylation of HER2 during the conditions of Cav-1 up-regulation in GC cells. We also found that the increase of Cav-1-induced phosphorylation of HER2 was blocked with the abrogation of Met. Since the Cav-1 was knocked down, the inactivation of HER2 was occurred prior to the decrease of Met expression. The importance of our finding is that Cav-1 may serve as a crucial linker for the positive crosstalk between Met and HER2 signaling pathway, leading to the cisplatin resistance in GC.

That being said, animal model of cisplatin resistance should be explored and used in further stduies to identify the chemoresistance-promoting role of Cav-1 in gastric cancer. Clinial studies should be conducted to reveal the interaction between Cav-1 and Met in the cisplatin-resistant patients. Collectively, our results provide a novel molecular mechanism that the up-regulation of Cav-1 prevents gastric cancer cells from the cisplatin-induced apoptosis, and it may function as a crucial regulator in the cisplatin-resistance of GC cells by activating the WNT/β-catenin signaling pathway and enhancing the Met-HER2 positive feedback. Targeted therapies against the activation of Cav-1-dependent WNT signaling might be developed to improve the extremely poor prognosis of gastric cancer patients with cisplatin-resistance.

## Data Availability Statement

All datasets generated for this study are included in the article/supplementary material.

## Author Contributions

BL, ZC, and LF conceived the concept. XW and ZC designed the study. XW, CD, YF, KH, and BZ performed the experiments. XW and LF wrote the paper. All authors have seen the manuscript and approved to submit to your journal.

### Conflict of Interest

The authors declare that the research was conducted in the absence of any commercial or financial relationships that could be construed as a potential conflict of interest.

## Abbreviations

Cav-1: Caveolin-1
WNT: wingless-type MMTV integration site
GC: gastric cancer
FZD: frizzled
LRP5/6: low-density lipoprotein receptor-related protein
Dvl: Disheveled
CDDP: cisplatin
Met: MET proto-oncogene receptor tyrosine kinase
HER2: Human epidermal growth factor receptor-2.
